# Potential applications of cyanobacteria: *Spirulina platensis* filtrates and homogenates in agriculture

**DOI:** 10.1007/s11274-019-2653-6

**Published:** 2019-05-27

**Authors:** K. Godlewska, I. Michalak, P. Pacyga, S. Baśladyńska, K. Chojnacka

**Affiliations:** 1 Department of Horticulture, The Faculty of Life Sciences and Technology, Wrocław University of Environmental and Life Sciences, pl. Grunwaldzki 24A, 50-363 Wrocław, Poland; 2 Department of Advanced Material Technologies, Faculty of Chemistry, Wrocław University of Science and Technology, Smoluchowskiego 25, 50-372 Wrocław, Poland; 3 Department of Design Fundamentals and Fluid-Flow Machinery, Faculty of Mechanical and Power Engineering, Wrocław University of Science and Technology, Na Grobli 15, 50-421 Wrocław, Poland

**Keywords:** Filtrate, Foliar spray, Homogenate, Biostimulant, Seed coating, Seed soaking, *Spirulina platensis*

## Abstract

In the present paper, products obtained from a blue-green microalga *Spirulina platensis* filtrate (applied for seed soaking and for foliar spray) and homogenate (used for seed coating) were tested in the cultivation of radish. Their effect on length, wet mass, multielemental composition and the greenness index of the radish leaves was examined. Multi-elemental analyses of the algal products, and radish were also performed using inductively coupled plasma-optical emission spectrometry (ICP-OES). The best soaking time, concentrations of filtrate and doses of homogenate were established. The longest and heaviest plants were observed for homogenate applied at a dose of 300 µL per 1.5 g of seeds and 15% of filtrate applied as foliar spray. The highest chlorophyll content was found in the group treated with 100 µL of homogenate and 5% of filtrate. In the case of soaking time, the longest plants were in the group where seeds were soaked for 6 h, but the heaviest and greenest were after soaking for 48 h. The applied algal products increased the content of elements in seedlings. Obtained results proved that algal extracts have high potential to be applied in modern horticulture and agriculture. The use of *Spirulina*-based products is consistent with the idea of sustainable agriculture that could help to ensure production of sufficient human food to meet the needs of rising population and protection of the environment.

## Introduction

Recently, intense research on microalgal formulations useful in cultivation of plants is observed. Algae are characterised by higher productivities than terrestrial plants and can be used for the production of valuable products for plants, including fertilizers (Wuang et al. 2016). Cyanobacteria can play a crucial role in the sustainable agriculture that contributes to the soil fertility, crop growth and yield and improvement of the environmental quality (Singh et al. 2016; Osman et al. 2016). The use of dried cyanobacteria to inoculate soil in order to increase its fertility is called “algalization”. Mishra and Pabbi (2004) showed that the addition of algae to the soil can increase rice yield by 15–20% in field experiments. Cyanobacterial biomass is also known to improve soil physicochemical characteristics such as water-holding capacity and mineral status of the degraded soils (Singh et al. 2016). Many cyanobacteria [e.g., *Nostoc muscorum*, *Nostoc humifusum*, *Anabaena oryzae* and *Wollea* sp. (Hegazi et al. 2010)] are capable of using atmospheric nitrogen as a source of nitrogen (nitrogen fixation) (Bhowmik et al. 2010; Mishra and Pabbi 2004; Singh et al. 2016) which may reduce the amount of synthetic nitrogen fertilizers used in agriculture (Bhowmik et al. 2010; Hegazi et al. 2010). In the work of Hegazi et al. (2010) it was suggested that 1/4 or 1/2 of the recommended dose of nitrogen mineral fertilizer could be reduced by using some species of cyanobacteria capable of nitrogen fixation. *Spirulina* formulations can be treated as biostimulants of plant growth, which according to the definition presented by du Jardin (2015) are “any substances or microorganisms applied to plants with the aim to enhance nutrition efficiency, abiotic stress tolerance and/or crop quality traits, regardless of its nutrients content”.

*Arthrospira platensis* (*Spirulina platensis*) can be used as a rich source of macro- and micronutrients for plants—for example vitamins, amino acids, polypeptides, phytohormones (gibberellins, auxins, cytokinins), antioxidants and compounds with antibacterial and antifungal properties (Bhowmik et al. 2010; Osman et al. 2016; Nawrocka et al. 2017). Despite their unique chemical composition, microalgae are not as often used for agricultural purposes as macroalgae (seaweeds). This can result from the availability of seaweed biomass—it is usually abundant in many marine and freshwater reservoirs, whereas microalgae are usually cultivated in the artificial conditions. Therefore, they constitute more expensive source of biomass for the production of biostimulants of plant growth.

Literature review on the application of *Spirulina* in plant cultivation is presented in Table 1. In most cases, *Spirulina* was applied directly to the soil or was added in the form of the algal suspension. Plants biofortified with the macro- and micronutrients of cyanobacterial origin can be used as novel, functional food preventing the malnutrition (Tuhy et al. 2015; Mala et al. 2017). Mala et al. (2017) used *S. platensis* as a fertilizer for agronomic biofortification of *Amaranthus dubius* (the red spinach) with carbohydrates, proteins, essential macronutrients, micronutrients and vitamin A. Post-extraction residues after supercritical CO_2_ extraction of *S*. *platensis* enriched with Zn(II), Cu(II) Mn(II) ions using biosorption were used as NPK fertilizer bio-components to biofortify maize in field trials with these micronutrients (Tuhy et al. 2015). Anitha et al. (2016) used *S. platensis* as a biofortified material to enhance zinc level in cultivars of a vegetable amaranth (*Amaranthus gangeticus*), mung bean (*Phaseolus aureus*) and tomato. Enzymatic hydrolysates of *S. Platensis* contain polyamines (e.g., spermine) obtained by the decarboxylation of algal L-amino acids, which are known to promote plant growth (Mógor et al. 2018). Different approach was proposed by Osman et al. (2016) who used *S. platensis* as a natural safener (instead of chemical) against harmful effects of fusilade herbicide on faba bean plant (faba bean seeds were primed in a *S. platensis* suspension before cultivation).

**Table 1:** Literature review on the application of *Spirulina* in plant cultivation

Species	Experimental conditions	Effect of *Spirulina* products	References
*Spirulina platensis*	Field; plots of 4 × 4 *Amaranthus dubius* *S. platensis*—5 g per plot	Germination in control was 82% and 95% in 0.005% of *S. platensis* Leaves fortified with vitamin A Total chlorophyll content was ~ 4 times higher and protein content more than 6 times higher in the experimental group than in the control Antioxidant activity of leaves from the experimental group was ~ 2.5 times higher than in the control group	Mala et al. (2017)
*Spirulina platensis* post-extraction residues after supercritical CO_2_ extraction enriched with Zn(II), Cu(II) Mn(II) ions by biosorption	Field; 21 m^2^ plots Maize Each group: fertilization with NPK (MgS) Experimental groups with bio-components: (a) *Spirulina*-Zn from 118 kg ha^−1^ (100% of the requirement) to 235 kg ha^−1^ (200%) (b) *Spirulina*-Mn from 89 kg ha^−1^ (100%) to 179 kg ha^−1^ (200%) (c) *Spirulina*-Cu from 12.5 kg ha^−1^ (100%) to 25 kg ha^−1^ (200%)	Grain yield for *Spirulina* 100% (Zn + Mn + Cu)—7.2 tones ha^−1^ was higher than in control group—untreated (6.2 tones ha^−1^) The highest content of micronutrients in plants was observed for maize grains fertilized with *Spirulina* 150%: preparation 2.15 mg kg^−1^ for Cu, 7.07 mg kg^−1^ for Mn and 29.0 mg kg^−1^ for Zn	Tuhy et al. (2015)
Enzymatic hydrolysate of *Spirulina platensis* and lyophilisate	Foliar application of hydrolysates Bioassay for cytokinin-like effect was conducted using cucumber cotyledons (*Cucumis sativus* L.), for auxin-like effect was performed using mung bean (*Vigna radiata* L.) and *C. sativus* Bioassays with lyophilised and hydrolysed *S. platensis* (2, 4 and 6 h of hydrolysis) Field trials with hydrolyzed *S. platensis* on lettuce (*Lactuca sativa*)	The hydrolysates had a cytokinin-like effect in the bioassay *S. platensis* lyophilized and its hydrolysates (2, 4, and 6 h of hydrolysis) did not promote root emission in *V. radiata* Hydrolysates influenced better *C. sativus* cotyledons fresh weight when compared with lyophilised *S. platensis*, cytokinin standard and control group Leaf area of lettuce seedlings was the highest in the group treated with foliar applications of hydrolysate (hydrolysis for 4 h) Foliar applications of the 4 h reaction hydrolysate the most effective promoted growth and the content spermine (polyamine) by 64% in the lettuce leaves	Mógor et al. (2018)
*Spirulina platensis* suspension	Faba bean (*Vicia faba*: Giza 843) were sterilized in sodium hypochlorite solution (1%), washed with distilled water, then primed in distilled water (as control) or 1% *S. platensis* suspension for 12 h Pots in a green house	Amelioration of the harmful effects of the herbicide on the antioxidant enzymes, reduction of the lipid peroxidation and proline content of the plant (*S. platensis* suspension induces the biosynthesis of some amino acids which could protect or act as a safener) Enhancement in protein and amino acid levels of root and shoot	Osman et al. (2016)
*Spirulina* suspension	Field; plot size 2.7 m × 3.5 m Mung bean (*Vigna radiata* (L.) Wilczek) seeds were presoaked in *Spirulina* suspensions (1, 3, 5, 7, 9 g L^−1^) for 6 h and in water as a control group	The highest number of branches, clusters, pods and seed yield per plant were obtained for the suspension with concentration 7 g L^−1^	Aung (2011)
*Spirulina platensis*	Field Seeds of *Amaranthus gangeticus*, *Phaseolus aureus* and tomato (variety-PKM1) Experimental groups: (a) Seeds soaking in different concentrations of *Spirulina* (5, 10, 15, 20, 25, 30 g in 100 mL of water) (b) Seeds soaking in *Spirulina* hydrolysate (5 g of *Spirulina* in 100 mL of water) at different time (1, 2, 3, 4, 5 h and overnight) (c) Spirulina in combination with biofertilizers, chemical fertilizer, organic fertilizer and vermicompost in various proportions (ratio: 25:75; 50:50; 75:25) (d) Foliar spray with different concentrations of *Spirulina* (25, 50, 75, 100 g in 5 L of water)	*Spirulina* increased the zinc level in all three plants when compared with the control group The combination of *Spirulina* and biofertilizer in the ratio of 75:25 showed the highest zinc level in *A. gangeticus* (77 mg kg^−1^ dry mass; d.m.) The combination of *Spirulina* and organic manure in the ratio of 50:50 showed the highest zinc level in *Phaseolus aureus* (54 mg kg^−1^ d.m.) Soaking of seeds for 2 h resulted in the highest zinc content (5.28 mg kg^−1^ d.m.) in tomato cultivar	Anitha et al. (2016)
*Spirulina platensis*, *Spirulina maxima*	Dose: 500 and 1000 mg of *Spirulina* per kg of soil Tested plants: *Phaseolus aureus* and *P. mungo* in clay pots	Better results in terms of plant shoot length were observed for a dose 1000 mg kg^−1^ For both doses plant shoot length was higher than in the control group (difference statistically significant) Chlorophyll and protein content of the grains was comparable in all tested groups (these parameters were not improved significantly)	Bhowmik et al. (2010)
Mixture of *Nostoc muscorum, Nostoc humifusum, Anabaena oryzae, Wollea* sp.*, Phormedium* and *Spirulina platensis*	Field experiment common bean (*Phaseolus vulgaris* L.; var. Nebraska) Application methods of algal mixture (a) Seed coating with air dried algal biomass (b) Soil drench application with algal culture suspension (c) The combination of seed coating and soil drench in the presence of 50 and 75% of the recommended dose for bean of nitrogen mineral fertilizers	The performance of bean plants: fresh and dry weight/plant, plant height, number of leaves/plant, leaf area/plant was enhanced by cyanobacteria The combined application of cyanobacteria—method (c) with using 75% of the recommended chemical N fertilizer was found to be the best treatment for enhancing plant growth Data demonstrated that dry application method is better than drench method Addition of algae can reduce the amount of chemical nitrogen either by 50% (dry and drench) without affecting seed yield characters	Hegazi et al. (2010)
*Spirulina platensis* (cultivation of *Spirulina* platensis in fish water—aquaculture wastewater remediation—removal of ammonia and nitrate)	Pot experiments on arugula (*Eruca sativa*), bayam Red (*Amaranthus gangeticus*), and pak choy (*Brassica rapa* ssp. *chinensis*) Application methods of algae (a) *Spirulina* (5 g pot^−1^) (b) *Spirulina* (5 g pot^−1^) plus chemical fertilizer (0.3 g pot^−1^ week^−1^)	*Spirulina* enhanced plant growth (number of leaf, plant height, root length, chlorophyll content, fresh weight, dry weight) of all tested vegetables (especially arugula, bayam red and pak choy), when compared to the controls (lack of additives, chemical fertilizers) The germination of pak choy improved significantly in terms of seedlings' dry weight	Wuang et al. (2016)
*Spirulina maxima* extracts	Extracts obtained from *S. maxima* with distilled water, methanol and hexane cultivation of *Vigna radiata* and *Oryza sativa* var. *Japonica*	Distilled water and methanol extracts inhibited germination Hexane extract showed no effect on seed germination *S. maxima* extract has the potential as a natural herbicide	Sornchai et al. (2014)
Formulation containing *Spirulina* extract obtained by supercritical fluid extraction	Germination tests on wheat seeds seed coating with 3 doses (8, 14 and 20 μL per 1 g of seeds) of formulation	The best results of the sprout growth were achieved for seeds coated with 8 μL of *Spirulina* sp. formulation Coating resulted in the increase of the biomass yield (by ~ 13%) and the sprout height (by ~ 7%) when compared to the water	Dmytryk et al. (2014)

In this study, the blue-green microalga, *S. platensis* was used as a raw material for the production of filtrate (applied for seed soaking and for foliar spray) and homogenate (used for seed coating). Natural products were tested in the cultivation of radish (*Raphanus sativus*) in the germination tests. Seed industry is nowadays recognized as a crucial sector that can increase the productivity of crops (Singh et al. 2015). Therefore, the aim of the research was to examine the effect of the obtained bioproducts on the morphological indicators of radish seedlings (length of above-ground biomass, weight of wet biomass, chlorophyll content in the biomass), as well as on the biofortification of the above-ground radish biomass with micro- and macroelements derived from microalga.

## Materials and methods

### Chemicals and microalgae biomass

The 69% nitric acid, spectrally pure (Suprapur) was purchased from Merck KGaA (Darmstadt, Germany) and dried biomass of *Spirulina platensis* from WB Im-und Export W. Beringer & Co. GmbH (Görmin/Böken; Germany) in 2016.

### Production of preparations

Homogenate was prepared by a suspension of dry *S. platensis* in deionised water (in a ratio 1:10) and mixing (Thermomix; Vorkwerk Ltd., Poland) at 37 °C for 40 min. (500 rpm). The obtained solution was centrifuged for 20 min. (4600 rpm) (Heraeus Megafuge 40, rotor TX-750, Thermo Scientific, Waltham, MA, USA). Supernatant was separated and treated as an algal filtrate (F)—100% and then (1) foliarly applied to the sprouts as an aqueous solution at different concentrations (5, 7, 10, 15, 20 and 25%, *v/v*). The 15% concentration was used for (2) seeds soaking for different time spans–1, 3, 6, 12, 18, 24, 36 and 48 h. The remaining solid residue, treated as an algal homogenate (H)–100%, was diluted with deionised water (1:1) and used for (3) seeds coating (doses–100, 300, 500, 700 µL per 1.5 g of radish seeds). This treatment was performed using vortex-type shaker for 10 min. The concentrations were selected on the basis of our previous studies (Michalak et al. 2017).

## Germination tests–Petri dish tests

**Fig. 1:**
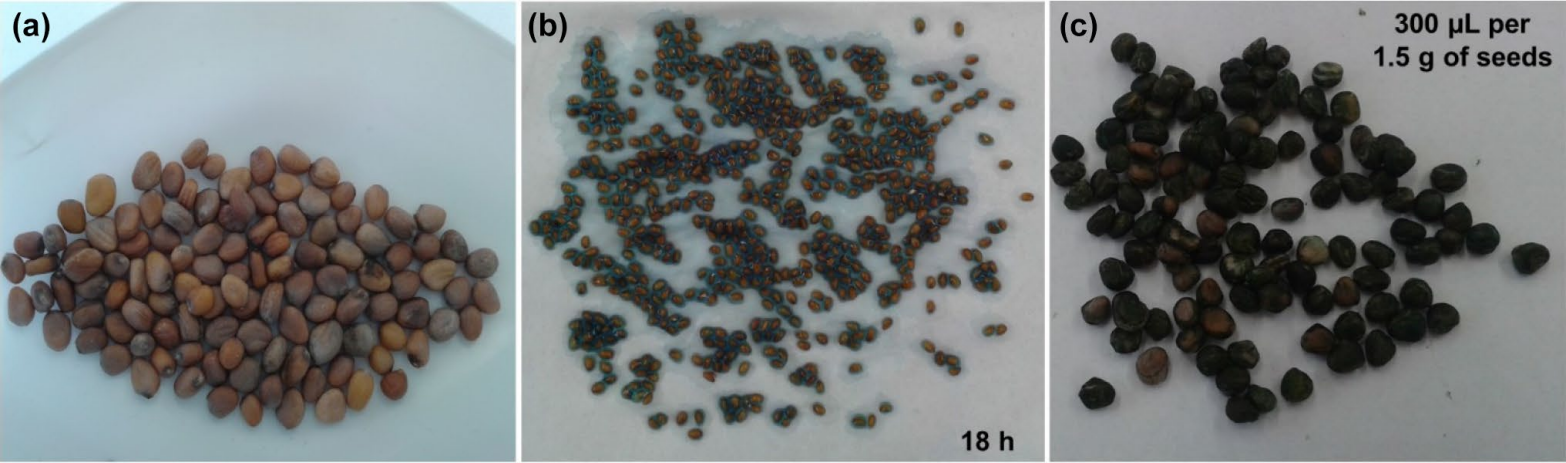
Radish seeds without pre-treatment (**a**), radish seeds soaked (**b**) and treated with *Spirulina* homogenate (**c**)

The phytotoxicity of the algal formulations was evaluated in the germination tests. Experiments were carried out under controlled conditions: 21 ± 1 °C, constant humidity and 12/12 h light/dark photoperiod, limiting the risk of abiotic and/or biotic stress. As a model plant, radish (*Raphanus sativus* 'Caro', TORSEED, Toruń, Poland) was chosen. Radish seeds—without pre-treatment, as well as soaked in the *Spirulina* filtrate and treated with *Spirulina* homogenate are presented in Fig. 1. Experiments were conducted on Petri dishes, in 3 replications for each group in standardized conditions using Jacobsen apparatus (Laborset, Łódź, Poland) according to the international norm (International Rules for Seed Testing, 2011—International Seed Testing Association (Bassersdorf, Switzerland)). On each Petri dish (diameter 85 mm), 25 seeds were placed on moistened cotton wool (5 g; Lohmann and Rauscher company, Rengsdorf, Germany). Next, each dish was sprayed three times with 5 mL of algal filtrate (after 6th, 8th and 10th day). The control group (C) was consistently watered with deionised water and sprayed with commercial biostimulant (CB; Asahi SL, SUMIN, Wargowo, Poland). The humidity of the cotton wool was kept at a constant level. After 13 days cultivated plants were collected and analyzed.

## Tested parameters after germination tests and analytical methods

The efficacy of *Spirulina*-based preparations was evaluated on the basis of plant height, fresh and dry mass, greenness index of the leaf, multielemental composition, and compared to series treated with deionised water (C) and commercial biostimulant (CB). The results are presented as an average from three measurements of 25 seedlings collected from each Petri dish. The greenness index of the radish leaf was determined using SPAD-502 Chlorophyll Meter (Konica Minolta, Japan). The plant biomass was dried at temperature of 50 °C (dryer Wamed SUP-30, Warsaw, Poland).

In order to determine the multielemental composition of *Spirulina* products, as well as radish mineralization of the samples was performed. Organic matter was removed from plant samples or *Spirulina*-based preparations with the use of 2.5 or 5 mL, respectively of 69% spectrally pure nitric acid (Suprapur, Merck KGaA, Darmstadt, Germany) in Teflon bombs in a microwave oven (Milestone S.r.l., Sorisole, Italy). After mineralization, samples were diluted with demineralized water (Millipore Simplicity, Darmstadt, Germany) to 25 or 50 g, respectively. Digested samples underwent a multielemental analysis using ICP-OES iCAP (6500 Duo, Thermo Scientific, Waltham, MA, USA) in the laboratory of Multielemental Analyses at Wrocław University of Science and Technology, which is accredited by ILAC-MRA and Polish Centre for Accreditation (No. AB 696).

### Statistical analysis

The results were elaborated statistically using *Statistica* ver. 12 (StatSoft Polska Ltd., Cracow, Poland). Appropriate statistical tests were used: the Shapiro–Wilk test (normality of the distribution of experimental results), the Brown-Forsythe test (for normal distribution and homogeneity of variances), the (RIR) Tukey test (for a comparison of all pairs of means following one-way ANOVA) and the Kruskal–Wallis test (for the verification if the distribution of the results was other than normal). Results were considered significantly different when *p* < 0.05.

## Results

### Multielemental composition of *Spirulina platensis* products

In the present study, *S. platensis* was homogenized and then centrifuged. The liquid part—supernatant was used as a filtrate/extract for plants spraying (as a biostimulant of plant growth) and for seeds soaking before sowing, whereas the solid residue was used for seeds coating also before their sowing. Table 2 presents a multielemental composition of *S. platensis* products—filtrate and homogenate and commercial biostimulant of plant growth. It is worth noting that macroelements—K, Mg, P, S occurred in larger quantities in a homogenate than in filtrate, whereas microelements—Cu, Fe, Ni and Zn in an algal filtrate. The content of all studied elements was much higher in *Spirulina* products than in the commercial biostimulant. Therefore, microalga products not only improve plant growth and development but also enhance the mineral composition of cultivated plants.

**Table 2:** Multielemental composition of *Spirulina platensis* products—filtrate, homogenate and commercial product (mg L^−1^)

Element/wavelength (nm)		*S. platensis* filtrate	*S. platensis* homogenate	Commercial biostimulant (CB)
Al	308.215	197.2 ± 29.6	756.1 ± 113.4	< 1.0
Ca	315.887	3042 ± 608	3141 ± 628	40.76 ± 31.38
Cr	267.716	< 0.3	3.236 ± 0.485	< 0.3
Cu	324.754	3.364 ± 0.505	3.947 ± 0.592	0.3407 ± 0.1979
Fe	259.940	353.6 ± 53.0	1464 ± 293	< 0.4
K	766.491	38,687 ± 7737	9641 ± 1928	3.037 ± 0.356
Mg	285.213	4588 ± 918	2701 ± 540	5.120 ± 2.689
Mn	257.61	74.40 ± 11.16	46.64 ± 7.00	< 0.25
Na	588.995	13,375 ± 2675	3753 ± 751	1328 ± 18
Ni	231.604	2.289 ± 0.343	8.096 ± 1.214	< 0.1
P	213.618	18,687 ± 3737	7336 ± 1467	< 10
S	181.972	7234 ± 1447	6789 ± 1358	< 10
Si	251.611	154.0 ± 23.1	119.1 ± 17.9	2.545 ± 0.879
Zn	213.857	18.74 ± 2.81	19.42 ± 2.91	1.499 ± 0.790

## The effect of *Spirulina* products on morphological indicators of radish seedlings

**Fig. 2:**
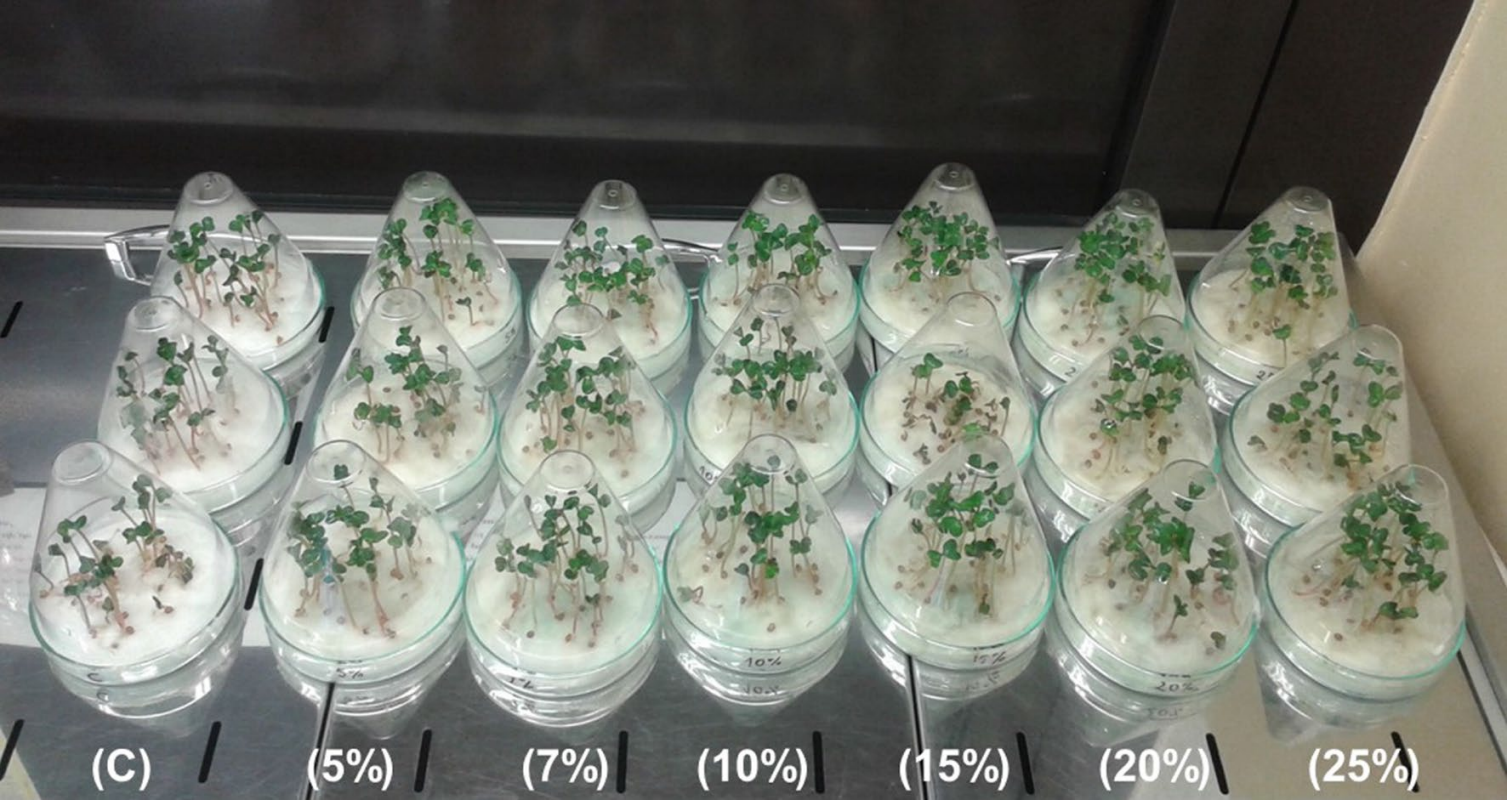
Germination tests of radish treated with *Spirulina* filtrate

The effect of homogenate (doses—100, 300, 500, 700 µL per 1.5 g of radish seeds) used for seeds coating, 15% filtrate used for seeds soaking (time span—1, 3, 6, 12, 18, 24, 36, 48 h) and filtrate used for foliar spray (concentrations—5, 7, 10, 15, 20 and 25%) on the length of the above-ground biomass, wet and dry mass was examined. The example of the radish germination tests with *Spirulina* filtrates is presented in Fig. 2.

### The effect of *Spirulina* products on the above-ground radish length.

After 13 days of the experiment, plants from each replication (N = 3) were collected and the length was determined for all *Spirulina*–based products and control groups. Both algal and commercial biostimulant showed a beneficial effect on the length of model plant. In order to verify the statistically significant differences between the tested groups, analyses were performed using STATISTICA software.

**Fig. 3:**
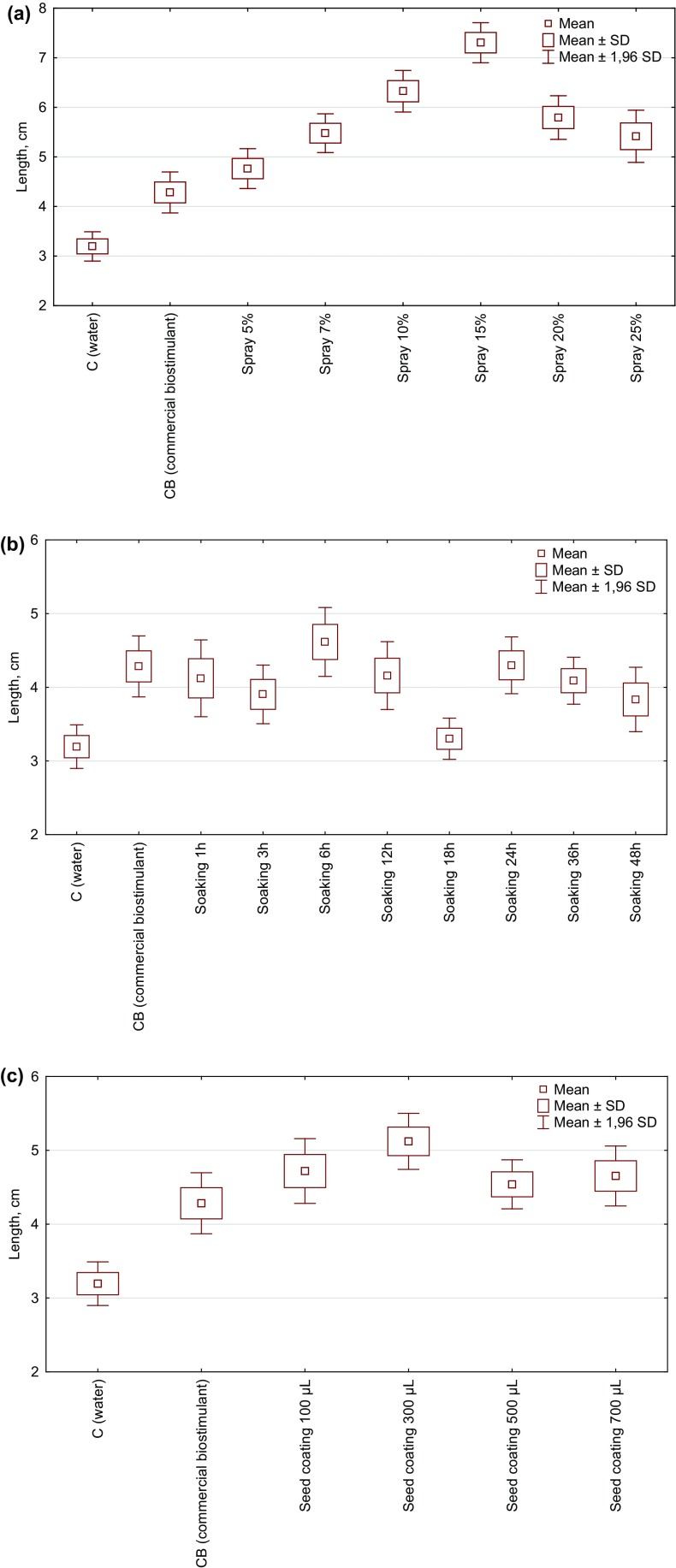
The effect of **a** a foliar spray of *Spirulina* filtrate, **b** seeds soaking in *Spirulina* filtrate (1–48 h), **c** seeds coating in *Spirulina* homogenate (100–700 µL per 1.5 g of seeds) on the radish length (cm; N = 3)

The effect of a foliar spray of *Spirulina* filtrate on the radish length is presented in Fig. 3a. It was observed that with the increasing concentration of the preparation, the length of plants increased. However, a decreasing biostimulating activity was observed above 15% concentration of the filtrate. The application of 15% solution influenced radish length in the highest extent, which was 129 and 71% longer than in C and CB, respectively. The statistically significant differences (for *p* < 0.05) were observed between the control group (C) and all the tested concentrations of biostimulants, but not for CB. Statistically significant differences were observed between CB and extracts at concentrations of 7, 10, 15 and 20%.

The effect of seeds soaking in 15% *Spirulina* filtrate on the above-ground biomass length is presented in Fig. 3b. Soaking time in 15% algal filtrate showed varying degree of a stimulating effect on the radish growth. All *Spirulina* treatments had a beneficial influence on the radish growth in comparison with the control group (C). The best time for seeds soaking proved to be 6 h where plants were higher when compared with C by 44.5% and CB by 7.7%. A longer time of seeds treatment in filtrate showed to be ineffective. The statistically significant differences were found between the control group and soaking for 6, 24 and 36 h and CB, and between CB and seeds soaking for 18 h.

The effect of seeds coating in *Spirulina* homogenate on the above-ground radish length is presented in Fig. 3c. All *Spirulina*–based homogenates showed greater biostimulating properties than the commercial product. The highest plants were obtained in a group treated with 300 µL of homogenate per 1.5 g of seeds. In this group, plants were higher respectively by 60.5 and 20% in comparison with C and CB. The statistically significant differences were observed between the control group and all the tested homogenate’s doses and the commercial biostimulant. In comparison with CB, the difference was noted only for 300 µL.

### The effect of *Spirulina* products on the fresh mass of radish

**Fig. 4:**
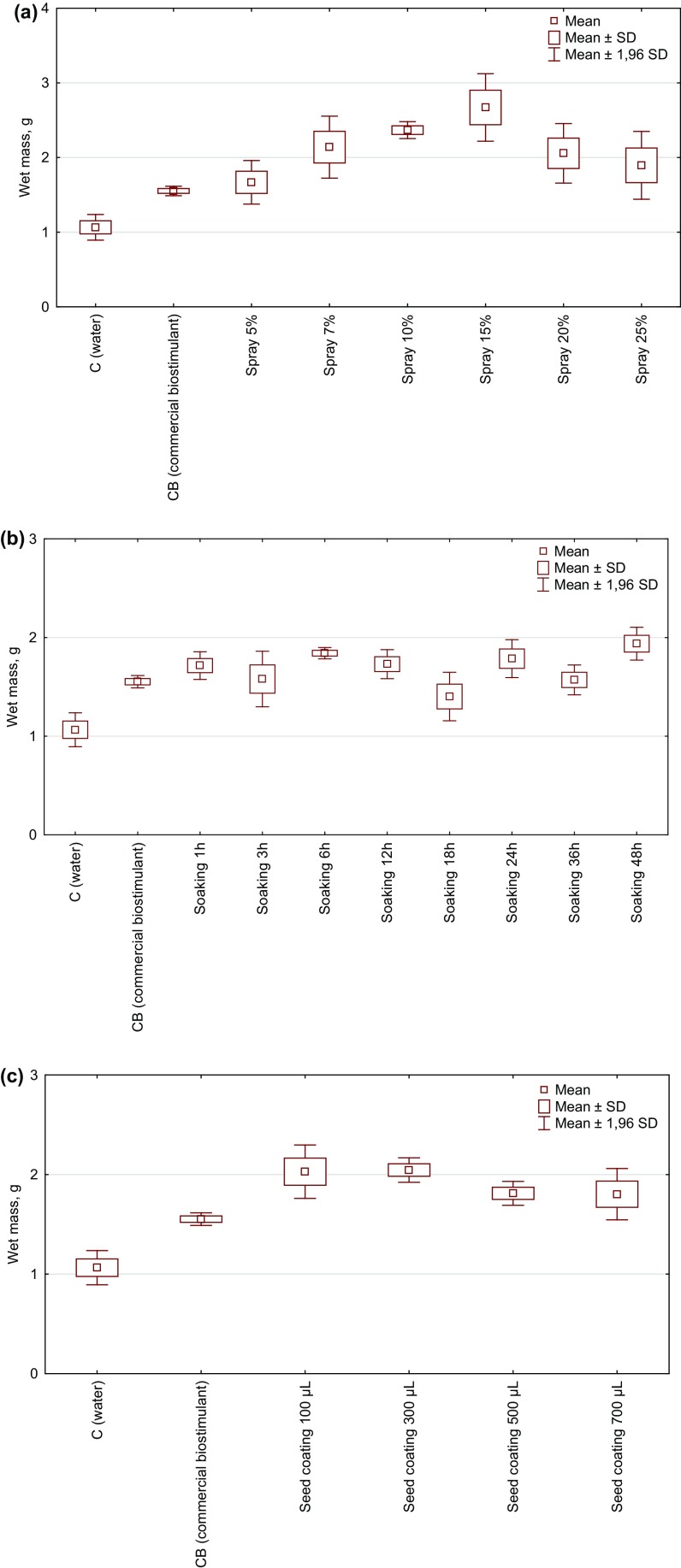
The effect of **a** a foliar spray of *Spirulina* filtrate, **b** seeds soaking in *Spirulina* filtrate (1–48 h), **c** seeds coating in *Spirulina* homogenate (100–700 µL per 1.5 g of seeds) on the radish wet mass (g; N = 3)

First, the effect of a foliar spray of *Spirulina* filtrate on the fresh mass of radish was tested. As can be seen from Fig. 4a, for the 15% concentration of microalgal filtrate—plants were heavier by 151 and 72% than in the control and commercial biostimulant group, respectively. With the increasing concentration of filtrates, the fresh mass was decreasing. The statistically significant differences were reported between the groups treated with 10 and 15% filtrate and the control group.

The effect of seeds soaking in 15% *Spirulina* filtrate on the radish fresh mass is presented in Fig. 4b. Soaking of seeds showed stimulating effect on the plants fresh biomass. Generally, in all experimental groups, the plant mass was higher when compared with C and CB. The best result was observed for the longest time (48 h) of seeds soaking (increase by 81 and 25% when compared with C and CB, respectively). The lightest plants were in a group where seeds were soaked for 18 h (heavier by 31% than in C and lighter by 9.7% than in CB). The statistically significant differences were found between the control and experimental group (except 18 h) and commercial biostimulants. In comparison with CB, produced *Spirulina* extracts did not show any significant differences.

Finally, we tested the effect of seeds coating in *Spirulina* homogenate on the radish fresh mass. The results showed that the applied biostimulants positively influenced the fresh mass of radish (Fig. 4c). It could be noticed that the highest masses were in the groups treated with 300 and 100 µL of homogenate (92 and 90% heavier than in C; 32.3 and 31% heavier than in CB). The increasing dose of preparations (500 and 700 µL) emerged to be less effective (69 and 68% heavier than in C and 17 and 16% than in CB). The statistically significant differences were observed only between the control group and 300 µL of homogenate.

### The effect of *Spirulina* products on the greenness index of the radish leaf

**Fig. 5:**
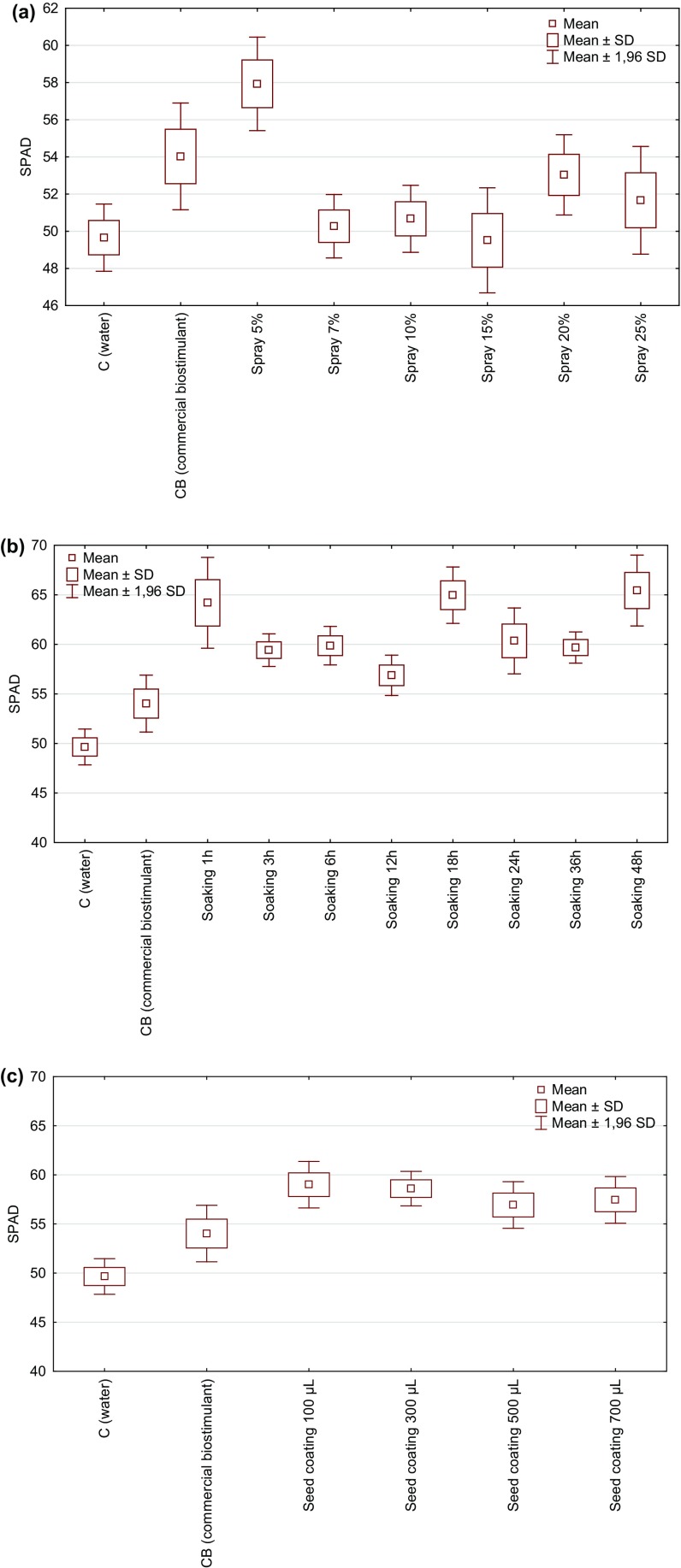
The effect of **a** a foliar spray of *Spirulina* filtrate, **b** seeds soaking in *Spirulina* filtrate (1–48 h), **c** seeds coating in *Spirulina* homogenate (100–700 µL per 1.5 g of seeds) on the greenness index of the radish leaf (N = 3)

The foliar application of *Spirulina* filtrate on radish seeds resulted in the increased content of green pigment (Fig. 5a). In the groups 15% < 7% < 10% < 25% the greenness index was statistically insignificant in comparison with the control group. The application of 5% filtrate statistically enhanced the greenness by 17 and 7.2% when compared to the control and the commercial product, respectively. Another statistically significant difference was observed in the group treated with 20% filtrate (6.8% higher when compared to C). No statistically significant differences were observed between the commercial product and the control group and tested filtrates.

The seeds soaking in a *Spirulina* filtrate increased the greenness index of the radish leaf in all the tested groups (Fig. 5b). The greenest leaves were noticed for 48 h of seeds soaking (32 and 20% more than in C and CB, respectively). The least biostimulating properties showed time of 12 h where the SPAD value was higher by about 15 and 4.8% in comparison with C and CB. The statistically significant differences were observed for all tested biostimulants (except 12 h and commercial product) when compared with the control. The soaking time—1, 18 and 48 h showed significant differences in comparison with CB.

The application of homogenates for seed coating resulted in the increased greenness index of the radish leaves (Fig. 5c). The highest chlorophyll content was noticed in a group treated with 100 µL of preparation (19 and 9.2% more than in C and CB, respectively) while the lowest with 500 µL (15% more than in C). The statistically significant differences were noted between water (C) and 100, 300, 700 µL of homogenates (not for CB). Among the tested *Spirulina* products, the highest greenness index of the radish leaves was recorded for the group with homogenate (100 µL/1.5 g of seeds).

## Multielemental composition of the above-ground biomass of radish

The effect of the different methods of the application of *Spirulina* products on the multielemental composition of radish was examined. *Spirulina* filtrate used as a foliar spray increased the content of plant micronutrients when compared with the control group (water)—Table 3. Boron and copper content generally increased with the increasing concentration of the algal filtrate (from 5 to 20%). Decrease was observed after the application of 25% *Spirulina* filtrate. The content of B in radish was 2.5 times higher in the group with 20% filtrate than in the control group, for Cu it was 30%. Iron content increased in the radish biomass with increasing concentration of *Spirulina* filtrate till the concentration of 20% (increase by 46% in relation to the control group). For 25% filtrate iron content decreased in the biomass, however it was higher than in the control group. Manganese content in all experimental groups was higher than in the control group, but the best results were obtained for 20% filtrate (increase by 34%). The application of 20% filtrate resulted also in the highest nickel and zinc content in radish—increase by ~ 5.5 times and 55% when compared with the water.

**Table 3:** The effect of foliar spray of *Spirulina* filtrate on the radish elemental composition (mg kg^−1^ d.m.; N = 3)

Group		Commercial biostimulant (CB)		H_2_O (control)		Concentration of *Spirulina* filtrate											
						5%		7%		10%		15%		20%		25%	
Element/wavelength (nm)		Mean	SD	Mean	SD	Mean	SD	Mean	SD	Mean	SD	Mean	SD	Mean	SD	Mean	SD
Al	308.215	30.22	10.67	23.33	5.89	33.04	5.35	30.69	8.44	35.61	8.06	32.10	6.64	44.98	9.95	23.56	5.51
Ca	315.887	8688	1036	6538	1051	9637	442	9910	71	8650	946	10,568	2111	11,075	1268	7209	1942
Cr	267.716	1.023	0.260	0.5124	0.2200	0.6522	0.2750	1.073	0.817	0.8646	0.4653	0.3962	0.3366	1.328	0.679	0.8410	0.3084
Cu	324.754	11.32	1.82	8.927	0.898	11.53	1.09	11.56	0.10	11.37	1.03	11.16	3.24	11.60	0.98	8.759	1.894
Fe	259.94	127.1	15.5	107.8	10.6	129.6	20.3	127.0	3.1	130.8	16.8	134.1	16.0	157.1	19.3	121.1	15.7
K	766.491	8694	913	8284	942	13,086	1547	12,127	2662	15,119	1369	19,970	4406	21,216	2978	16,077	3470
Mg	285.213	5255	359	4177	496	5853	410	5900	324	5663	592	6236	706	6886	738	5324	706
Mn	257.61	44.77	7.85	33.77	4.17	42.97	2.04	43.00	6.93	39.12	4.63	41.63	8.56	45.18	4.78	34.80	6.42
Na	588.995	3204	1108	1716	914	2777	605	1193	244	5035	897	2813	948	4175	2479	3159	994
Ni	231.604	12.73	5.07	4.729	1.826	10.83	7.95	0.7609	LOD	9.046	LOD	8.771	4.377	26.52	28.11	7.920	5.326
P	213.618	14,758	848	12,855	1407	15,997	1032	15,186	392	15,095	1302	16,951	2084	19,594	1755	15,532	2586
S	181.972	22,784	1409	19,588	1756	21,912	1587	23,175	677	22,373	2546	22,917	2620	24,709	2125	22,756	2705
Si	251.611	55.43	21.01	48.61	25.82	75.58	32.50	76.04	16.00	76.04	38.95	65.76	30.53	62.37	12.53	55.94	10.05
Zn	213.857	98.66	11.19	80.56	8.952	111.7	14.2	112.5	5.7	124.5	17.6	122.6	29.1	124.6	9.9	90.64	19.44

*LOD* below limit of detection

Concluding results on the multielemental composition of radish after the application of *Spirulina* filtrates it can be noted that the best effect was obtained for 20% filtrate. Higher filtrates concentrations, generally caused a decrease of microelements content in the radish biomass. Analogous results were obtained for macroelements content in the biomass of radish—Ca, K, Mg, P and S. Filtrate with the concentration of 20% most beneficially influenced their content in the biomass. Algal filtrates/extracts are known to be active at low concentrations (diluted as 1:1000 or more) (Crouch and van Staden 1993). It is worth noting that the content of micro- and macroelements in radish after application of 20% filtrate was higher than in the group with the commercial biostimulant. For microelements it was as follows: for Ni two times higher, Zn by 26%, Fe by 24%, B about 8% higher and slightly Cu by 2.5% and Mn by 1%. Much larger differences were observed for the content of macroelements—Ca was by 27% higher in the group with 20% filtrate than for CB, K was 2.5 times higher, Mg by 31%, P by 33% and S by 8.5%.

In the present study we also examined the effect of seed soaking in 15% *Spirulina* filtrate on the multielemental composition of radish. The content of elements in the cultivated radish is presented in Table 4. It can be noted that soaking of seeds in *Spirulina* filtrate for more than 24 h generally negatively influenced the content of micro- and macroelements in the biomass. The best results were obtained for seed soaking for 24 h. In the case of microelements, the visible differences between the experimental groups and the control group (water), as well as the group with commercial biostimulant were as follows: content of B was about 2.4 times higher (1 h) than in the control and by 6% than in CB; content of Cu was by 36% higher (24 h) than in the control and by 7% than in CB; content of Fe was by 17.5% higher (24 h) than in the control but slightly lower than in CB; content of Mn was by 31% higher (24 h) than in the control and slightly lower than in CB; content of Ni was about 2 times higher (1 h) than in the control but lower than in CB and content of Zn was by 38% higher (24 h) than in the control and by 12% than in CB.

**Table 4:** The effect of seeds soaking in *Spirulina* filtrate (1–48 h) on the radish elemental composition (mg kg^−1^ d.m.; N = 3)

Group		Commercial biostimulant (CB)		H_2_O (control)		Period of seeds soaking in *Spirulina* filtrate															
						1 h		3 h		6 h		12 h		18 h		24 h		36 h		48 h	
Element/wavelength (nm)		Mean	SD	Mean	SD	Mean	SD	Mean	SD	Mean	SD	Mean	SD	Mean	SD	Mean	SD	Mean	SD	Mean	SD
Al	308.215	30.22	10.67	23.33	5.89	22.94	1.17	23.90	3.58	22.36	5.32	24.89	2.66	22.02	3.40	24.21	6.28	24.75	4.69	13.01	7.52
Ca	315.887	8688	1036	6538	1051	7103	886	6870	536	7346	695	6938	241	6654	1137	7890	1375	7430	454	5279	3313
Cr	267.716	1.023	0.260	0.5124	0.2199	0.9220	0.3901	0.6637	0.2681	0.7035	0.7846	0.5189	0.1870	0.3932	0.1440	0.8941	0.2591	0.8158	0.5714	0.6506	0.5818
Cu	324.754	11.32	1.82	8.927	0.898	9.101	0.419	10.43	0.40	11.12	0.87	9.606	0.367	9.542	1.547	12.12	1.65	11.06	0.48	8.084	4.812
Fe	259.94	127.1	15.5	107.8	10.6	111.3	9.0	118.7	11.0	115.3	10.9	107.8	7.9	116.1	11.6	126.7	18.9	125.6	8.1	89.26	54.79
K	766.491	8694	913	8284	942	7759	569	8216	1231	8580	1490	7673	187	6620	556	7978	649	7967	372	5322	3243
Mg	285.213	5255	359	4177	496	4502	383	4815	480	5110	770	4568	105	4341	365	5219	538	4951	193	3613	2250
Mn	257.61	44.77	7.85	33.77	4.17	35.85	1.34	35.52	1.92	38.63	1.44	35.49	1.49	33.92	3.15	44.35	9.15	41.01	4.91	27.12	15.33
Na	588.995	3204	1108	1716	914	3567	493	4043	1611	4269	541	4345	694	2527	1047	3531	365	4969	2511	2822	1962
Ni	231.604	12.73	5.07	4.729	1.826	9.474	0.397	5.614	2.721	4.457	LOD	5.051	4.248	3.071	0.256	7.282	1.519	5.718	1.916	5.318	3.934
P	213.618	14,758	848	12,855	1407	13,014	1341	13,417	927	14,072	1256	13,377	426	13,018	1076	14,603	1024	14,332	567	10,562	6575
S	181.972	22,784	1409	19,588	1756	18,746	1316	18,917	1233	20,532	1769	19,329	1466	18,694	501	22,768	1828	20,311	2965	16,107	9844
Si	251.611	55.43	21.01	48.61	25.82	78.14	10.82	56.30	24.35	49.48	1.02	54.86	10.12	40.42	8.52	68.83	11.48	50.46	12.17	38.38	20.27
Zn	213.857	98.66	11.19	80.56	8.95	88.04	6.93	96.59	5.29	103.5	14.1	90.56	1.91	88.54	9.66	110.9	19.1	107.7	15.9	72.72	43.89

In the case of macroelements, it was noted that (for the best experimental groups) the content of Ca was by 21% higher (24 h) than in the control but lower than in CB; content of K was by 3.6% higher (6 h) than in the control but lower than in CB; content of Mg was by 25% higher (24 h) than in the control but slightly lower than in CB; content of P was by 14% higher (24 h) than in the control but slightly lower than in CB and the content of S was by 16% higher (24 h) than in the control but slightly lower than in CB.

The multielemental composition of the radish cultivated from seeds treated with *Spirulina* homogenate before sowing is presented in Table 5. Generally, in the case of microelements (B, Cu, Fe, Mn, Ni and Zn) the best results were obtained for the highest dose—700 µL (with the exception for B—100 µL and Ni—500 µL). The content of B was 2.6 times higher for the dose 700 µL and 6 times higher for 100 µL than in the control group (water) and by 12% higher and 2.8 times, respectively when compared with the commercial biostimulant (CB); the content of Cu was by 49% higher for the dose 700 µL than in the control and by 18% than in CB; the content of Fe was by 30% higher for the dose 700 µL than in the control and by 11% than in CB; the content of Mn was by 64% higher for the dose 700 µL than in the control and by 24% than in CB; the content of Ni was by 61% higher for the dose 700 µL and about 2 times higher for 500 µL than in the control but the content was lower when compared with CB and the content of Zn was by 47% higher for the dose 700 µL than in the control and by 20% than in CB. In the case of the macroelements content, the level of Ca and Mg was the highest in the group 700 µL, whereas K, P and S in the group—100 µL. The differences in their content in radish were as follows: Ca was by 53% higher for the dose 700 µL than in the control and by 15% than in CB; Mg was by 52% higher for the dose 700 µL than in the control and by 21% than in CB; K was by 28% higher for the dose 100 µL than in the control and by 22% than in CB; P was by 28% higher for the dose 100 µL than in the control and by 11% than in CB; S was by 29% higher for the dose 100 µL than in the control and by 10% than in CB. Summarizing the results concerning the elemental composition of radish cultivated from the seeds treated with *Spirulina* homogenate, a dose 700 µL per 1.5 g of seeds can be recommended for further research—pot experiments in a greenhouse and then field trials.

**Table 5:** The effect of seeds coating in *Spirulina* homogenate (100–700 µL per 1.5 g of seeds) on the radish elemental composition (mg kg^−1^ d.m.; N = 3)

Group		Commercial biostimulant (CB)		H_2_O (control)		Dose of *Spirulina* homogenate for seeds coating							
						100 µL		300 µL		500 µL		700 µL	
Element/wavelength (nm)		Mean	SD	Mean	SD	Mean	SD	Mean	SD	Mean	SD	Mean	SD
Al	308.215	30.22	10.67	23.33	5.89	221.2	113.9	39.66	18.56	27.73	2.47	35.89	3.53
Ca	315.887	8688	1036	6538	1051	9319	933	9026	751	9806	726	9994	703
Cr	267.716	1.023	0.260	0.5124	0.2200	1.067	0.679	0.9426	0.2964	0.9240	0.5532	0.9749	0.9225
Cu	324.754	11.32	1.82	8.927	0.898	12.15	0.64	12.36	0.91	11.83	1.09	13.31	1.15
Fe	259.94	127.1	15.5	107.8	10.6	133.0	8.9	131.5	9.5	134.9	11.5	140.7	3.2
K	766.491	8694	913	8284	942	10,595	1932	10,072	678	10,254	1060	9619	2764
Mg	285.213	5255	359	4177	496	5661	602	5574	183	6003	248	6366	385
Mn	257.61	44.77	7.85	33.77	4.17	42.86	4.10	50.90	9.93	52.50	2.95	55.54	6.66
Na	588.995	3204	1108	1716	914	4096	1147	4756	1523	3186	436	2587	334
Ni	231.604	12.73	5.07	4.730	1.826	4.332	LOD	4.267	3.272	10.24	LOD	7.623	4.489
P	213.618	14,758	848	12,855	1407	16,442	1634	15,763	451	15,428	723	16,367	1281
S	181.972	22,785	1409	19,588	1756	25,183	1932	22,718	2549	24,654	1337	24,866	1879
Si	251.611	55.43	21.01	48.61	25.82	72.57	16.16	79.15	18.54	62.64	5.05	64.94	3.21
Zn	213.857	98.66	11.19	80.56	8.95	117.8	9.9	110.1	8.6	118.4	15.6	118.7	10.5

To sum up, the highest content of micro- and macroelements in the radish was obtained for the following groups—seed soaking in 15% *Spirulina* filtrate for 24 h, seed coating at a dose of 700 µL of *Spirulina* homogenate per 1.5 g of seeds and foliar spray with 20% *Spirulina* filtrate. Among these treatments, the smallest effect on the elemental content of radish had seeds soaking. Taking into account the production process and then practical application in greenhouse or field experiments, foliar method seems to be the most efficient.

## Discussion

In the present paper we examined the biostimulant properties of *Spirulina* products applied in the radish cultivation. Radish was used as a model plant since it reaches quite quickly the mature state (13 days).

### The effect of *Spirulina* products on the radish length

All concentrations of *Spirulina* filtrates influenced statistically the length of the above-ground biomass. These results are in agreement with the literature data (Table 1). Michalak et al. (2016) studied the impact of 10% *Spirulina* extract, obtained by supercritical fluid extraction, applied in three different doses 1.0, 1.5, and 1.8 L ha^−1^ on winter wheat (variety *Akteur*). Authors found that this biostimulant did not influence significantly the plant length. Opposite results were published by Aghofack-Nguemezi et al. (2015) who studied the effect of *Spirulina platensis* aqueous extract on the growth parameters and development of tomato plants. Particularly, the foliar spraying of aqueous extracts (3%) increased the plant length by 19% and the diameter by 33%.

For seeds soaking in 15% *Spirulina* filtrate, all examined treatments (different soaking period) had a beneficial influence on radish growth in comparison with the control group. This confirms results obtained by other authors. Aung (2011) prepared different concentrations of *Spirulina* suspensions (1, 3, 5, 7, 9 g L^−1^) and soaked seeds of *Vigna radiata* for 6 h. The untreated seeds (a control group) were pre-soaked in a purified water. In the 10th week of cultivation, plants in all experimental groups were higher than in the control, while the highest concentration produced the maximum plant length (12% longer). It can be seen that with the increasing doses of suspensions, the plant length also increased. Wuang et al. (2016) investigated the effect of *Spirulina* inoculation on seed germination. Trials were conducted on three types of vegetables—Chinese Cabbage (*B. rapa* ssp. *chinensis*), Kai Lan (*Brassica oleracea alboglabra*) and White Crown (*B. rapa* ssp. *chinensis*, F1 hybrid). The treatments were made at various concentrations of microalga (2, 4, 6, 8 and 10 g L^−1^) in tap water and control (tap water only). The seeds were soaked in the solutions overnight before germination on a tissue towel. For Chinese Cabbage, the only improvement in shoot length was observed for 4 g L^−1^ (14% longer). In the case of Kai Lan, almost all dilutions showed biostimulating properties (except 8 g L^−1^) and the highest plants were after application of 2 g L^−1^ (18% longer). There were no significant improvements in the shoot length of White Crown.

*Spirulina* homogenate, similarly like *Spirulina* filtrate used as a foliar spray or for seed soaking stimulated the above-ground radish length. The results obtained by Dmytryk et al. (2014) also proved that microalga *Spirulina* could be used for seed coating. In their work the impact of algal formulations containing 10 and 25% of supercritical CO_2_ extracts from *Spirulina* sp. on *Triticum aestivum* ssp. *vulgare* variety *Zyta* was investigated. Different doses of extracts were used i.e.: for 10%—10, 20, 100 µL per 1 g of seeds and for 25%—8, 14, 20 µL per 1 g of seeds. The lower concentration of the product (10%) in a dose of 20 µL proved to be the most stimulating the plant growth (12% higher when compared with water), while in higher concentrations (25%)—8 and 14 µL were slightly better than the control (6% higher). In the work of Hegazi et al. (2010), the effect of the treatment of common bean (*Phaseolus vulgaris* L.) seeds with dry microalga under different nitrogen levels on the plant length was investigated. In the case of plant length, in the first year of research, addition of *Spirulina* was as effective as the application of 100% N and in the second year caused the increase in plant length by 12% for the combination with 75% N.

It is worth mentioning, that the seed treatment before sowing (including seed soaking and seed coating) can play an important role in the increasing of the crops productivity due to the maintenance of the seeds quality and the improvement of their germination (Singh et al. 2015). The positive effect of these treatments on plant growth can result also from the absorption of nutrients, protectants, growth regulators by seeds from the appropriate solutions for extended periods (Scott 1989, 1998). In the present study, seeds were immersed in *Spirulina* products, which can serve as a rich source of novel and biochemically active natural compounds. Cyanobacteria are known to contain a wide variety of compounds, which include 40% of lipopeptides (with cytotoxic (41%), antitumor (13%), antiviral (4%), antibiotics activity (12%) and the remaining 18% activities include anti-malarial, antimycotics, multidrug resistance reversers, antifeedant, herbicides and immunosuppressive agents), 5.6% of amino acids, 4.2% of fatty acids, 4.2% of macrolides and 9% of amides (Singh et al. 2008). *Spirulina platensis* used in the present study is rich in free and bound amino acids, including alanine, glycine, valine, leucine, isoleucine, asparagine, aspartic acid, glutamine, glutamic acid, lysine, arginine, histidine, phenylalanine, tyrosine, tryptophan, serine, threonine, methionine, cysteine and proline (Nawrocka et al., 2017). Osman et al. (2016) showed that *Spirulina* suspension can also induce the biosynthesis of proteins and amino acids in roots and shoots, which can act as a protector from harmful effect of herbicides. In the work of Nawrocka et al. (2017) it was also shown that *Spirulina platensis* contains also pigment—phycocyanin (266 ± 23 mg 100 g^−1^ d.m.), polyphenols, determined with Folin–Ciocalteu reagent (176 ± 5 mg 100 g^−1^ expressed as equivalents of gallic acid), vitamins such as α-tocopherol (2.43 ± 0.21 mg 100 g^−1^), γ-tocopherol (1.07 ± 0.21 mg 100 g^−1^) and ascorbic acid (18.37 ± 0.97 mg 100 g^−1^). All these compounds can be responsible for the biostimulant properties of *Spirulina* formulations. Mógor et al. (2018) showed also that the enzymatic hydrolysates of *Spirulina platensis* had a cytokinin-like effect which effectively promoted lettuce growth (Table 1).

### The effect of *Spirulina* products on the fresh mass of radish

In the present paper it was shown that all *Spirulina* products influenced positively the fresh mass of radish what confirms literature data for example (Hegazi et al. 2010; Tuhy et al. 2015; Wuang et al. 2016; Mógor et al. 2018) (Table 1). Aghofack-Nguemezi et al. (2015) presented that *Spirulina platensis* can affect the fresh biomass of aerial parts of tomato plants by 48% and the fruit biomass by 43% when compared with the control group (no treatment). Michalak et al. (2016) investigated the impact of microalgal extracts on the yield parameters of grain and mass of 1000 grains of winter wheat (variety *Akteur*). The grain yield was comparable in all tested groups (all doses of *Spirulina* ranged from 1 to 1.8 L ha^−1^, control and commercial biostimulants). The statistically significant differences were noted for the mass of 1000 grains. The highest mass was obtained for *Spirulina*—1.8 L ha^−1^, *Spirulina*—1.0 L ha^−1^, in comparison with the control group by approximately 16%. The lowest mass was observed for 1.5 L ha^−1^*Spirulina* extract (higher than in control approx. by 13%). In the work of Aung (2011), the effect of *Spirulina* biofertilizer suspension on the growth and yield of *Vigna radiata* was examined. Among all the concentrations (ranged from 1 to 9 g L^−1^ of *Spirulina* suspension), 7 g L^−1^ produced the maximum seed yield (238 g m^−2^) of *Vigna radiata* (L.). The second, highest yield was produced from 9 g L^−1^ treatment (229 g m^−2^). The yield of the control was found to be the lowest (172 g m^−2^). In the work of Mógor et al. (2018), the increase of the fresh biomass of cucumber was attributed to the cytokinin-like activity of *Spirulina* hydrolysates.

In the literature, the positive effect of algal homogenates on the growth of plants is known and confirms our results (Table 1). In the work of Dmytryk et al. (2014), the wet biomass of winter wheat was about 16% and 13% heavier after application of 20 µL of 10% and 8 µL of 25% formulation than for control group (water). Hegazi et al. (2010) observed that the microalga increased the fresh mass of common bean—for example in a combination with 75% N and seed coating with *Spirulina*, plants were heavier by 26% in the first year and by 22.5% in the second year in comparison with the control with higher dose of N (100%). This solution can reduce the amount of used chemical fertilizers.

The positive effect of *Spirulina* products on the length and fresh mass of radish is also beneficial from the medical point of view. It is worth emphasizing that both leaves and roots of radish have been used in various parts of the world as antiviral (e.g., against influenza virus), antimicrobial, antioxidant agents and also to treat cancer (Pérez Gutiérrez and Perez 2004). Radish leaves were also found to reduce intestinal glucose absorption (Banihani 2017).

### The effect of *Spirulina* products on the chlorophyll content in radish

As it was shown in the present study, *Spirulina* products can also increase the chlorophyll content in the leaves of germinated radish, especially after the application of 5% filtrate, soaking of seeds for 48 h and coating of seeds in 100 µL of homogenate. Dmytryk et al. (2014) stated that chlorophyll content in *Cucumis sativus* was affected by neither supercritical extract from *Spirulina* nor reference products when compared with the control groups. In the work of Hegazi et al. (2010), the effect of seed coating with *Spirulina* and the simultaneous application of nitrogen fertilizer on the chlorophyll content in leaves of common bean was presented. In the first year, the use of alga and appropriate doses (50 and 75%) of N resulted in a higher content of a green pigment (13 and 16.5% more, respectively) in comparison with 100% of N; whereas in the second year results were comparable. *Spirulina* applied to the soil can increase not only the chlorophyll content in the leaves, but also the content of proteins and amino acids (Bhowmik et al. 2010; Osman et al. 2016; Mala et al. 2017), vitamin A (Mala et al. 2017) and antioxidant activity of the cultivated biomass (Mala et al. 2017). Higher chlorophyll content in the leaves can also result from the higher leaf area due to the action of *Spirulina* hydrolysate (Mógor et al. 2018), or dry algal biomass used for seeds coating (Hegazi et al. 2010).

### Multielemental composition of the above-ground biomass of radish

Micro- and macroelements, as well as other nutrients present in *Spirulina* products play a major role in plant metabolism (e.g., physiological activities like cellular organization, protein and nucleic acid metabolisms) (Anitha et al. 2016). They influence not only growth and development of the cultivated plants, but also their chemical composition. Therefore, they can be useful and beneficial for human nutrition and health. In this work, *Spirulina platensis* products, rich in micro- and macroelements biofortified the above-ground biomass of radish with mineral elements. Their content was higher in the radish from experimental groups when compared with the control group. The presence of bioactive compounds (e.g., amino acids, carbohydrates, peptides) in biostimulants of plant growth such as *Spirulina* filtrate/homogenate can increase the content of minerals in the plant due to increased sink strength that influences the movement of substrates, including minerals, within the plant (Calvo et al. 2014). Biostimulants of plant growth improve also the mineral uptake by the well-developed root system (improved lateral root formation and increased total volume of the root system) and leaves (increased number of leaves per plant and their area) (Khan et al. 2009).

In the literature it was shown that the application of *Spirulina* products can increase the content of micro- and macroelements in the crops, fruits, vegetables. For example, Tuhy et al. (2015) recorded enrichment of maize with Zn, Mn and Cu derived from a solid *Spirulina* biomass applied as a micronutrient fertilizer. Anitha et al. (2016) examined the effect of different concentrations of *Spirulina* suspension in water (5, 10, 15 and 20 g L^−1^) on the content of zinc in the biomass of *Amaranthus gangeticus*, *Phaseolus aureus* and tomato plants. The results showed that *Spirulina* filtrate applied foliarly can help to accumulate essential substances (e.g., zinc) which are needed for plant growth. In the work of the same author, it was shown that soaking of seeds of *Amaranthus gangeticus*, *Phaseolus aureus* and tomato plants for 1, 2, 3, 4, 5, 25 h in the suspension of 5 g of *Spirulina* in 100 mL of water generally resulted in the enrichment of plants in zinc. For *Amaranthus gangeticus* the best soaking time was 4 h (54.5 mg kg^−1^), for *Phaseolus aureus*—3 h (50.8 mg kg^−1^) and for tomato plants—2 h (5.28 mg kg^−1^).

The biomass of radish biofortified with microelements can be used to prevent from so called “hidden hunger” which is referred to micronutrient deficiencies (Kennedy et al. 2003; Burchi et al. 2011). This is a global challenge to health, especially of vulnerable population like women and children all over the world. One of the strategies for addressing micronutrient malnutrition is fortification (including biofortification). A crucial is also dietary diversification and supplementation (Kennedy et al. 2003).

Summarizing, in the present study, we examined the effect of natural products (filtrates and homogenates) obtained from microalga *Spirulina platensis* in the cultivation of radish. Most of tested biostimulants increased the length of plants in comparison to control and commercial product. The longest aerial parts of radish were in the group treated with 15% of spray, soaked for 6 h in 15% of filtrate, and coated with 300 µL of homogenate per 1.5 g of seeds. In the case of wet mass, 15% of filtrate, 48 h of soaking and 300 and 100 µL of homogenate for coating proved to be the mostly stimulating. The highest concentration of chlorophyll was in groups sprayed with 5% of filtrate, soaked for 48 h and coated with 100 µL of product. It was found that taking into account the content of micro- and macroelements in the biomass of cultivated radish it was the highest in the following groups—seeds soaking for 24 h, seeds coating at a dose of 700 µL of homogenate per 1.5 g of seeds and foliar spray of *Spirulina* extract at a concentration of 20%. These applications resulted in the production of biofortified edible vegetables that can be beneficial to human health. For further research—greenhouse experiments and then field trials, *Spirulina* extract applied foliarly is highly recommended.
